# Association Between Balance Self-efficacy and Walking Ability in Those With New Lower Limb Amputations

**DOI:** 10.33137/cpoj.v5i1.36695

**Published:** 2022-01-11

**Authors:** C. Frengopoulos, Z. Zia, M.W.C Payne, R. Viana, S.W. Hunter

**Affiliations:** 1 Faculty of Health Sciences, University of Western Ontario, London, Ontario, Canada.; 2 Michael G. DeGroote School of Medicine, McMaster University, London, Ontario, Canada.; 3 Department of Physical Medicine & Rehabilitation, Parkwood Institute, London, Ontario, Canada.; 4 Department of Physical Medicine & Rehabilitation, Schulich School of Medicine & Dentistry, University of Western Ontario, London, Ontario, Canada.; 5 School of Physical Therapy, University of Western Ontario, London, Ontario, Canada.

**Keywords:** Amputation, Self-Efficacy, Balance Confidence, Walking Ability, Rehabilitation, PEQ

## Abstract

**BACKGROUND:**

A relationship between walking ability and self-efficacy has been demonstrated in various rehabilitation patient populations. In experienced prosthetic ambulators, walking ability is related to self-efficacy of balance, however, this relationship has not been quantified for those with newly acquired lower limb amputations (LLA).

**OBJECTIVES:**

To investigate the association between walking performance (objective) and self-reported walking abilities (subjective) on balance self-efficacy in those with LLA.

**METHODOLOGY:**

Cross-sectional study of 27 people (17 men; mean age=63.57±9.33) at discharge from inpatient prosthetic rehabilitation for first major unilateral LLA. Individuals completed 6m straight path walking and the L-Test under single- and dual-task conditions. The Prosthesis Evaluation Questionnaire (PEQ) was administered, and the Ambulation subscale provided subjective measures of walking ability. A single PEQ question on satisfaction with walking (16B) was also used as a proxy for subjective walking ability. The Activities-specific Balance Confidence Scale measured balance self-efficacy. Multivariable linear regression was used to evaluate the strength of association between walking ability (objective and subjective) and balance self-efficacy (dependent variable).

**FINDINGS:**

Walking velocity on the 6m straight path under single-task (p=0.011) and dual-task conditions (p=0.039), the single-task L-Test (p=0.035) and self-reported satisfaction with walking (p=0.019) were associated with self-efficacy of balance.

**CONCLUSIONS:**

Objective measures of walking ability that were independently associated with balance self-efficacy included straight path walking velocity under single and dual-task conditions and the single-task L-Test. Satisfaction with walking was also associated with balance self-efficacy. This highlights the interplay between physical and psychological factors during rehabilitation. More research in the area of self-efficacy and walking ability is needed to establish self-efficacy as a target during prosthetic rehabilitation for those with LLA.

## INTRODUCTION

Individuals encounter significant physical, psychological and social consequences following the loss of a limb.^1^ While the physical concerns of decreased mobility are paramount, there are also difficulties with community participation and body image that requires attention.^2^ Rehabilitation for lower limb amputation (LLA) is multidisciplinary and goals include restoring independent mobility and enhancing quality of life (QOL).^3^ Performance on objective measures of mobility is often considered to be the gold standard for evaluating rehabilitation progress or success.^4^ However, subjective assessments can also be considered as they may provide insight for perceived mobility in scenarios not tested in clinical environment. There is an established trend in walking ability following prosthetic rehabilitation for LLA; the largest improvements in self-reported functional mobility occur between 6 weeks and 4 months post-surgery, after which time function plateaus.^5^

While mobility and function improve during rehabilitation, the LLA population may experience difficulties with gait that persist long-term.^6,7^ Falls are a particular concern in this population, as over half of those with LLA fall at least once annually.^8,9^ After a fall, older individuals without LLA may limit their walking due to impact on self-efficacy and a fear of falling.^9^ However, walking is crucial for social participation and maintaining QOL in those with LLA.^10^ As such, the impact of psychological factors on walking ability in this population have been investigated. Research indicates that factors such as self-efficacy may impact upon the rehabilitation goals of social activity and community integration for those with LLA.^11,12^

Self-efficacy is an individual's belief in their ability to perform a certain task.^13^ Developing self-efficacy may be done through observing successful peers or through verbal persuasion from credible individuals, such as physicians.^13,14^ However, the strongest sources of self-efficacy are mastery experiences; other sources are generally weak and are likely to deteriorate.^14,15^ Mastery experiences are the successful completion of a specific task.^15^ These experiences can build upon each other as an individual accomplishes progressively more difficult activities.^15^ As such, the mastery experience of ambulating with a prosthetic device would be expected to strongly impact a person's sense of competence and self-efficacy.

Research has demonstrated a positive relationship between walking performance and self-efficacy following a stroke,^16^ in those with knee osteoarthritis,^17,18^ for those with diabetes and peripheral arterial disease,^19^ and in individuals with multiple sclerosis.^20^ However, the experience following LLA is different to these populations as the latter have previous mastery experiences from which they derive their self-efficacy. At the onset of rehabilitation, all tasks relating to the prosthetic device are novel. Prosthetic training provides a series of mastery experiences for new skills related to the use of a prosthesis, allowing those with LLA to develop self-efficacy. Individuals also receive encouragement from clinicians during their time in prosthetic rehabilitation and are able to observe fellow patients. Levels of self-efficacy during this rehabilitation process have not been reported, however, for more experienced prosthetic ambulators, walking ability is related to balance self-efficacy.^12^

Physical and psychological factors are both important considerations for rehabilitation following LLA. For the psychological factor of self-efficacy to be addressed as a target during the rehabilitation process, a better understanding of the relationship between walking ability and self-efficacy is needed. To quantify the relationship between self-efficacy of balance and walking ability in those with LLA, self-reported measures of ability and objective walking performance must be carried out. The objective of this study was to investigate the association between walking performance (objective) and self-reported walking ability (subjective) on balance self-efficacy in those with LLA at discharge from inpatient prosthetic rehabilitation. It is hypothesized that at this stage in the rehabilitation process straight path walking and complex path walking under single-task conditions, as well as patient satisfaction with walking, will be related to balance self-efficacy.

## METHODOLOGY

### Design and Participants

This was a cross-sectional study of individuals completing inpatient prosthetic rehabilitation for first major LLA. Participants were recruited from the Regional Amputee Rehabilitation Program at Parkwood Institute in London, Ontario between March 2016 and April 2017. The study was approved by the University of Western Ontario Ethics Board, and by the Lawson Health Research Institute Clinical Resources Impact Committee. All individuals provided written informed consent prior to participating in the study.

Individuals participating in an inpatient rehabilitation program for their first major, unilateral LLA were recruited prior to discharge. LLA at the level of transtibial or above were considered major amputations; these were selected as the represent the most common levels seen in prosthetic rehabilitation programs. All individuals were new prosthetic ambulators as they were fitted with a prosthesis upon commencement of inpatient rehabilitation. Admission criteria to inpatient rehabilitation include achievable rehabilitation and prosthetic goals, medically stable and sufficiently conditioned to undergo a training program, ability to learn and retain new skills, and emotionally and socially (housing, funding, outside responsibilities, etc.) prepared to participate. Training involved prosthetic limb fitting, limb care and management, and safe skill learning to progress from transfers to gait training with the use of various gait aids. Eligibility criteria for the current study were: ≥50 years of age, functional use of English and ability to walk 10m without assistance from another person. Exclusion criteria included any physical problem that significantly limited movement or presence of severe depression. Those were severe depression were excluded as depression can negatively impact performance on cognitive tasks requiring attention and visuospatial abilities, especially in older adults.^21^ A total of 27 participants were recruited during the study period.

The following demographic and medical history information was obtained: age, sex, height, weight, level of amputation, etiology of amputation, time since amputation, use of walking aid at discharge, level of education, medications, comorbidities, history of falls in the past 12 months, global cognitive status as measured by the Montreal Cognitive Assessment (MoCA).

### Objective Measures of Walking Ability

The L-Test is a complex walking task that evaluates functional mobility for those with LLA.^22^ Testing was carried out under single- and dual-task conditions with standardized instructions.^22,23^ Using standard instructions, participants began sitting on an armless chair and upon the word “go” rose to standing and walked 3 meters, turned 90 degrees, walked 7 meters, turned 180 degrees, and walked the same path to return to a seated position. Patients walked at their usual pace and were timed to the nearest 100th of a second.

A 6m straight path assessed gait velocity (cm/s) under single- and dual-task conditions. Participants walked at their usual pace along the GAITRite^®^ System electronic walkway (CIR Systems, Franklin, NJ). One-meter acceleration and deceleration zones were provided to ensure only steady state walking was captured. Assessment of gait velocity using the GAITRite^®^ System was performed separately to the walking assessment using the L-Test.

### Subjective Measures of Walking Ability

The Prosthesis Evaluation Questionnaire (PEQ) is a self-administered tool that evaluates prosthetic related QOL.^24^ It consists of 9 stand-alone subscales including Ambulation, Appearance, Frustration, Perceived Response, Residual Limb Health, Social Burden, Sounds, Utility and Well Being. The Ambulation subscale was used to assess self-perceived walking ability (questions 13A, 13B, 13C, 13D, 14E, 14F, 14G, 14H).^24^ The following standalone question relating to participant satisfaction (question 16B) was also used: “Over the past four weeks, rate how satisfied you have been with how you are walking”. Questions utilize a 100mm Visual Analog Scale (VAS) from “cannot” (0) to “no problem” (100). Higher scores indicate a more positive response.^24^ The complete PEQ was administered to participants.

### Measure of Balance Self-Efficacy

The ABC Scale is a 16-item tool that evaluates balance confidence, or self-efficacy of balance.^25^ Participants rate how confident they are in performing activities without losing their balance or becoming unsteady, from 0% to 100%. A mean of the 16 items gives the overall score, with higher scores indicating better balance self-efficacy.^25^ The scale has good test-retest reliability and internal consistency in the LLA population.^26^

### Testing Protocol

Single-task (walking test alone) and dual-task (walking task paired with cognitively demanding task) walking assessments were performed in this study. Demographic and clinical information was collected first. Following this, walking tasks were demonstrated by a research assistant. Participants then completed single-task assessments and received a 5-minute break before completing dual-task testing. Only one trial per walking test was completed. Dual-task testing using a cognitively demanding task was used to more closely approximate real-world walking.^27^ The task chosen to represent this increased cognitive load was serial subtraction by 3's, starting from a randomly selected number between 100 and 150. Participants were instructed to count aloud as they walked, and responses were recorded. No instructions on task prioritization were given. All assessments occurred within 48 hours of discharge. Patients used their usual gait aids to perform walking tests. A standardized protocol, based on Hunter et al.,^23^ was used during testing.

### Statistical Analysis

Variables were calculated as means and SDs or frequencies and percentages, as appropriate. Multivariable linear regression was used to evaluate the strength of association between walking ability (objective and subjective) and balance self-efficacy (dependent variable). Regression diagnostics were performed to ensure assumptions for linear regressions were met. To generate the most parsimonious model, analysis was adjusted for age, level of amputation, number of comorbidities and number of medications. These variables were selected based on the literature and their clinical significance.^2,28,29^ Due to the small sample size of the current study, no subgroup analysis based on level of amputation was performed. Significance level was set to p<0.05. Statistical analysis was performed using the IBM SPSS Statistics version 24.0 (IBM Corporation, Armonk, NY).

## RESULTS

Demographics are summarized in **Table 1**. Twenty-seven participants were included in the study. Many participants (77.8%, n=21) had transtibial amputations; the remainder of the participants had transfemoral amputations. Almost 2/3rds of the study participants were male (63%, n=17). The mean balance self-efficacy rating using the ABC scale was 69.48 ± 14.50, with scores ranging from 35.63 to 89.69.

**Table 1:** Demographic and study characteristics of older adults with first major, unilateral lower limb amputation at discharge from prosthetic rehabilitation. (*n=27*).

Characteristics	Values
Age (years)	63.6 ± 9.3
Sex (men)	17 (63.0%)
Body Mass Index (kg/m^2^)	27.4 ± 5.4
Amputation level (n, % transtibial)	21 (77.8%)
Time between amputation and discharge (days)	142.6 ± 74.6
Fall in the previous 12 months? (n, % yes)	19 (70.4%)
Self-reported number of falls	2.1 ± 2.7
Montreal Cognitive Assessment	25.7 ± 2.9
**Primary Etiology of Amputation (n, %):**	
Diabetes Mellitus	17 (63.0%)
Peripheral Vascular Disease	4 (14.8%)
Diabetes Mellitus and Peripheral Vascular Disease	1 (3.7%)
Trauma, Cancer or Other (clotting disorders, infection, etc.)	5 (18.5%)
Number of medications	10.2 ± 4.6
Number of comorbidities	5.4 ± 2.2
**Walking aid use at discharge (n, %):**	
Single Cane	3 (11.1%)
Two Canes	3 (11.1%)
Forearm Crutches	1 (3.7%)
Standard Walker	1 (3.7%)
Rollator Walker	19 (70.4%)

Results from objective and subjective measures of walking ability are presented in **Table 2**. After adjusting for confounders, single-task and dual-task walking velocity along the 6m straight path were both found to be independently associated with self-efficacy of balance (**Table 3**). For every 1cm/s increase in walking velocity along a 6m path, balance self-efficacy increases by 0.32 for single-task (p=0.011) and 0.28 for dual-task conditions (p=0.039).

**Table 2:** Results of objective and subjective measures of walking ability at discharge from prosthetic rehabilitation. (*n=27*). Abbreviations: PEQ, Prosthesis Evaluation Questionnaire.

Variables	Mean ± SD
**Objective:**	
6m straight path, single task walking velocity (cm/s)	48.98 ± 25.26
6m straight path, dual task walking velocity (cm/s)	42.54 ± 22.86
L-Test, single-task walking time (seconds)	79.89 ± 53.01
L-Test, dual-task walking time (seconds)	97.90 ± 70.40
**Subjective:**	
PEQ Ambulation subscale	68.22 ± 19.09
PEQ 13A: Walking ability w/prosthesis	81.82 ± 16.32
PEQ 13B: Ability to walk in close spaces w/prosthesis	76.33 ± 24.80
PEQ 13C: Walk up-stairs w/prosthesis	78.16 ± 20.30
PEQ 13D: Walk down stairs w/prosthesis	77.72 ± 21.48
PEQ 14E: Walk up hill w/prosthesis	54.76 ± 33.17
PEQ 14F: Walk down-hill w/prosthesis	53.71 ± 32.14
PEQ 14G: Walk sidewalks w/prosthesis	77.83 ± 30.88
PEQ 14H: Walk on slippery surfaces w/prosthesis	45.43 ± 30.77
PEQ question 16B - Satisfaction with walking	88.44 ± 11.80

Single-task performance on the L-Test and satisfaction with walking were also independently associated with self-efficacy of balance (**Table 3**). For every one second decrease in performance on the L-Test balance self-efficacy increases by 0.17 (p=0.035), and for every 1-point increase in satisfaction with walking self-efficacy increases by 0.60 (p=0.019). No associations between the other objective or subjective measures and balance self-efficacy were found.

**Table 3:** Results of multivariable linear regression for the association of objective (6m straight path and L-test walking tests) and subjective (PEQ) measures of walking ability on self efficacy (Activities-specific Balance Confidence Scale). Abbreviations: β, regression coefficient; CI, confidence interval; PEQ, Prosthesis Evaluation Questionnaire. *Adjusted for age, level of amputation, number of comorbidities, number of medications.

Gait Variable	Unadjusted β (95% CI)	Adjusted β (95% CI)*
**Objective:**		
**6m straight path:**		
Single task walking velocity (cm/s)	**0.37 (0.19, 0.55), p<0.001**	**0.32 (0.08, 0.56), p=0.011**
Dual task walking velocity (cm/s)	**0.35 (0.13, 0.57), p=0.003**	**0.28 (0.02, 0.54), p=0.039**
**L-Test:**		
Single-task walking time (seconds)	**-0.17 (-0.26, -0.09), p<0.001**	**-0.17 (-0.33, -0.01), p=0.035**
Dual-task walking time (seconds)	**-0.11 (-0.18, -0.04) p=0.005**	-0.08 (-0.20, 0.04), p=0.164
**Subjective:**		
PEQ Ambulation Subscale Total Score	**0.35 (0.07, 0.63), p=0.016**	0.22 (-0.08, 0.52), p=0.140
PEQ 16B: Satisfaction with walking	**0.80 (0.41, 1.18), p<0.001**	**0.60 (0.11, 1.08), p=0.019**

## DISCUSSION

This study is the first to investigate associations between subjective and objective measures of walking ability and balance self-efficacy in those with LLA at discharge from prosthetic rehabilitation. We identified that straight path walking velocity under single and dual-task conditions, time to complete the single-task L-Test and self-reported satisfaction with walking were associated with balance self-efficacy at discharge from rehabilitation. This indicates that performance on both objective and subjective measures of walking ability influence the reported balance self-efficacy of individuals with LLA at this point in the prosthetic rehabilitation process.

The relationship between self-efficacy and motor learning is circular. As individuals gain self-efficacy they create more challenging goals; striving for these goals benefits motor learning and performance, leading to further goal setting.^30^ According to Bandura, the more mastery experiences that one has, the higher they perceive their ability;^15^ this perception of one's capabilities may be more related to performance than physical ability itself.^14,15^ This is an important consideration for individuals with LLA, as levels of balance self-efficacy tend to be low.^12,31^ The results from the current study highlights the relationship between walking ability and self-efficacy of balance at a single point in the rehabilitation journey. Further research is needed to fully understand the longitudinal relationship between self-efficacy, goal setting and walking ability for those with LLAs.

The 6m walk was the simplest measure performed by participants. In contrast, the L-Test is more complex as it incorporates transfers and turning,^22^ and dual-task testing provides an additional cognitive challenge to mimic real-world multi-tasking.^27^ As individuals progress through prosthetic rehabilitation, they gain self-efficacy through mastery of simple tasks, external coaching from expert clinicians and vicarious experiences of observing fellow patients.^13–15^ Confidence in performing more complex tasks develops later.^15^ The dual-task L-test is the most complex task that individuals were asked to perform in the current study. Therefore, it is possible that participants have not yet developed balance self-efficacy relating to this task. It is expected that individuals will continue to develop self-efficacy of balance through mastery experiences of more difficult walking tasks as they begin to ambulate in the community.^5^ As self-efficacy is activity specific, a task-specific intervention to improve self-efficacy may be a new target for interventions to improve walking ability for those with LLA. Task-oriented balance and walking interventions have been demonstrated to improve self-efficacy for individuals following a stroke.^32^

Predictors of walking ability following LLA have been widely described in the literature.^2,33^ Previously identified factors tend to refer to physical or objective findings such as level of amputation, age, physical fitness and etiology of amputation,^2,33^ and have not included the influence of psychological factors. However, Hamamura et al.^34^ did identify motivation to walk as a predictive factor for prosthetic rehabilitation in older adults with LLA. Previous research has also highlighted that while objective measures of walking ability improve following discharge from rehabilitation, subjective measures of walking ability do not.^31^ Self-efficacy and motivation, along with other psychological factors, may help to explain why subjective measures of walking ability do not improve. The current study adds to the literature and highlights the relationship between the psychological factor of balance self-efficacy and walking ability for those with LLA. More research on the relationship between psychological factors and their effects on subjective measures of walking ability is needed.

## Study Limitations

These results represent individuals with unilateral LLA at discharge from inpatient rehabilitation. More time with a prosthesis may impact self-efficacy of balance and the relationships described. The individuals in this study were >50 years of age, so results may not be relevant for younger cohorts or those with fewer comorbidities.

Transtibial and transfemoral levels of amputation were both represented in the current study, however no subgroup analysis based on level of amputation was performed due to small sample size. Also, for those with transfemoral amputations, the type of knee componentry was not recorded. Therefore, the current study is not able to comment on the impact of level of amputation or prosthetic components on the relationship between balance self-efficacy and walking ability. Analysis based on level of amputation should be considered for further studies.

Also, the objective walking measures used in the current study only examine short walking distances. Therefore, the association between objective measures of walking ability and balance self-efficacy cannot be generalized to longer walking measures or measures of endurance. Finally, the current study has a small sample size, which may be underpowered to detect the changes in all variables measured. In future, larger sample sizes should be used to confirm the results.

## CONCLUSION

This study demonstrates the interplay between physical and psychological factors during rehabilitation for LLA. Straight-path walking velocity under single and dual-task conditions, performance on the L-Test and self-reported satisfaction with walking are independently associated with self-efficacy of balance. However, the complex task of completing the dual-task L-Test was not found to be associated. Further research regarding other factors that may be associated with or influence self-efficacy is needed in the LLA population.

## DECLARATION OF CONFLICTING INTERESTS

The authors have nothing to disclose.

## AUTHOR CONTRIBUTION

Each author has made an equal contribution to the manuscript in the following areas: (1) concept or design of the work, or acquisition, analysis or interpretation of data; (2) drafted or critically revised the article; (3) approved the version to be published; (4) participated sufficiently to take public responsibility for appropriate portions of the content.

## SOURCES OF SUPPORT

This work was supported by the St. Joseph's Healthcare Foundation Cognitive Vitality and Brain Health Seed Funding Opportunity in London, Ontario, Canada. The funding body had no involvement in the conduct of the study.

## ETHICAL APPROVAL

The study was approved by the University of Western Ontario Ethics Board, and by the Lawson Health Research Institute Clinical Resources Impact Committee. All individuals provided written informed consent prior to participating in the study.
